
JOURNAL INFORMATION
==============================
NLM Title Abbreviation: Can Med Educ J
Journal ID: Can Med Educ J
NLM Journal ID: 101560935
Journal ID: CMEJ

Canadian Medical Education Journal

EISSN: 1923-1202
Publisher: University of Saskatchewan

ARTICLE INFORMATION
==============================
PMCID: PMC6500504
Article ID: CMEJ-10-e257
Article version: 1
Subjects: Abstracts

Poster Group Abstracts

Electronic publication date: 2019 Apr 3
Publication date: 2019 Apr
Volume: 10
Issue: 2
First page: e257
Last page: e293
Copyright year: 2019
License: This is an Open Journal Systems article distributed under the terms of the Creative Commons Attribution License (http://creativecommons.org/licenses/by/2.0) which permits unrestricted use, distribution, and reproduction in any medium, provided the original work is properly cited.
License URL: https://creativecommons.org/licenses/by/2.0/

==============================
JOURNAL INFORMATION
==============================
NLM Title Abbreviation: Can Med Educ J
Journal ID: Can Med Educ J
NLM Journal ID: 101560935
Journal ID: CMEJ

Canadian Medical Education Journal

EISSN: 1923-1202
Publisher: University of Saskatchewan

ARTICLE INFORMATION
==============================
Subjects: Pa - 1 – 1

Reducing Medical Student Performance Anxiety through the Pre-clerkship Residency Exploration Program (PREP)

Alysha Roberts Dalhousie University

Kavita Raju Dalhousie University

Thomas Sebastian Haupt Dalhousie University

Todd Dow Dalhousie University

Mike Smyth Dalhousie University

J Thomas Toguri Dalhousie University

Electronic publication date: 2019 Apr 3

JOURNAL INFORMATION
==============================
NLM Title Abbreviation: Can Med Educ J
Journal ID: Can Med Educ J
NLM Journal ID: 101560935
Journal ID: CMEJ

Canadian Medical Education Journal

EISSN: 1923-1202
Publisher: University of Saskatchewan

ARTICLE INFORMATION
==============================
Subjects: Pa - 1 – 2

Do offers of admission to regional medical campuses impact an applicant's decision to decline their acceptance to medical school?

Rosalind Bihun McMaster University

Margo Mountjoy McMaster University

Andrew Costa McMaster University

Graham Campbell McMaster University

Electronic publication date: 2019 Apr 3

JOURNAL INFORMATION
==============================
NLM Title Abbreviation: Can Med Educ J
Journal ID: Can Med Educ J
NLM Journal ID: 101560935
Journal ID: CMEJ

Canadian Medical Education Journal

EISSN: 1923-1202
Publisher: University of Saskatchewan

ARTICLE INFORMATION
==============================
Subjects: Pa - 1 – 3

Increasing Diversity Amongst Future Physicians - MD Admissions Initiative for Diversity and Equity

Emily Fong University of Alberta

Alex Wong University of Alberta

Tibetha Kemble University of Alberta

Electronic publication date: 2019 Apr 3

JOURNAL INFORMATION
==============================
NLM Title Abbreviation: Can Med Educ J
Journal ID: Can Med Educ J
NLM Journal ID: 101560935
Journal ID: CMEJ

Canadian Medical Education Journal

EISSN: 1923-1202
Publisher: University of Saskatchewan

ARTICLE INFORMATION
==============================
Subjects: Pa - 1 – 4

Improving medical student education in pediatrics through participation in Teddy Bear Clinics

Stephanie Chan McMaster University

Nathasha Dias McMaster University

Amanda Leslie McMaster University

Electronic publication date: 2019 Apr 3

JOURNAL INFORMATION
==============================
NLM Title Abbreviation: Can Med Educ J
Journal ID: Can Med Educ J
NLM Journal ID: 101560935
Journal ID: CMEJ

Canadian Medical Education Journal

EISSN: 1923-1202
Publisher: University of Saskatchewan

ARTICLE INFORMATION
==============================
Subjects: Pa - 1–5

Development and implementation of an Intellectual and Developmental Disability curriculum for first-year medical students at the University of Toronto

Maggie McCann University of Toronto

Kristen McFadyen University of Toronto

Olivia Spohn University of Toronto

Janet Vogt Surrey Place Centre

Barry Isaacs Surrey Place Centre

Alvin Loh Surrey Place Centre

Fok-Han Leung University of Toronto

Electronic publication date: 2019 Apr 3

JOURNAL INFORMATION
==============================
NLM Title Abbreviation: Can Med Educ J
Journal ID: Can Med Educ J
NLM Journal ID: 101560935
Journal ID: CMEJ

Canadian Medical Education Journal

EISSN: 1923-1202
Publisher: University of Saskatchewan

ARTICLE INFORMATION
==============================
Subjects: Pa - 1 – 6

Development of spaced repetition materials for undergraduate medical histopathology education

Jeremy Dick University of British Columbia

Karen Pinder University of British Columbia

Electronic publication date: 2019 Apr 3

JOURNAL INFORMATION
==============================
NLM Title Abbreviation: Can Med Educ J
Journal ID: Can Med Educ J
NLM Journal ID: 101560935
Journal ID: CMEJ

Canadian Medical Education Journal

EISSN: 1923-1202
Publisher: University of Saskatchewan

ARTICLE INFORMATION
==============================
Subjects: Pa - 1 – 7

Exploring international experiences of climate change in medical education

George T. Kitching Western University

Adrina Zhong Western University

Orianna Mak McMaster University

Marissa Ley Dalhousie University

Farnaz Javadian University of British Columbia

Electronic publication date: 2019 Apr 3

JOURNAL INFORMATION
==============================
NLM Title Abbreviation: Can Med Educ J
Journal ID: Can Med Educ J
NLM Journal ID: 101560935
Journal ID: CMEJ

Canadian Medical Education Journal

EISSN: 1923-1202
Publisher: University of Saskatchewan

ARTICLE INFORMATION
==============================
Subjects: Pa - 1 – 8

Standing Up to Block: Validating a Novel Ob/Gyn LIC Curriculum at the University of Toronto

Alexandra Davidson University of Toronto

Rajiv Shah University of Toronto

Anita Shah McMaster University

Lindsay Shirreff University of Toronto

Eliane Shore University of Toronto

Electronic publication date: 2019 Apr 3

JOURNAL INFORMATION
==============================
NLM Title Abbreviation: Can Med Educ J
Journal ID: Can Med Educ J
NLM Journal ID: 101560935
Journal ID: CMEJ

Canadian Medical Education Journal

EISSN: 1923-1202
Publisher: University of Saskatchewan

ARTICLE INFORMATION
==============================
Subjects: Pa - 2 – 1

The Community Health and Social Medicine Incubator (CHASM): A Pilot of Student Led Social Change

David-Dan Nguyen McGill

Julie de Meulemeester McGill

Kacper Niburski McGill

Gordon Best McGill

Koray Demir McGill

Electronic publication date: 2019 Apr 3

JOURNAL INFORMATION
==============================
NLM Title Abbreviation: Can Med Educ J
Journal ID: Can Med Educ J
NLM Journal ID: 101560935
Journal ID: CMEJ

Canadian Medical Education Journal

EISSN: 1923-1202
Publisher: University of Saskatchewan

ARTICLE INFORMATION
==============================
Subjects: Pa - 2 – 2

Naloxone kits at AEDs: A case-study of medical student driven advocacy in London, Ontario

Eric Mitchell Western University

James Riggs Western University

Rebecca Barnfield Western University

Trystan Nault Western University

Rajas Tipnis Western University

Electronic publication date: 2019 Apr 3

JOURNAL INFORMATION
==============================
NLM Title Abbreviation: Can Med Educ J
Journal ID: Can Med Educ J
NLM Journal ID: 101560935
Journal ID: CMEJ

Canadian Medical Education Journal

EISSN: 1923-1202
Publisher: University of Saskatchewan

ARTICLE INFORMATION
==============================
Subjects: Pa - 2 – 3

Medical Student Reflections on a Personalized Resource List

Leshawn Benedict University of Toronto

Kevin Si University of Toronto

Fok-Han Leung University of Toronto

Electronic publication date: 2019 Apr 3

JOURNAL INFORMATION
==============================
NLM Title Abbreviation: Can Med Educ J
Journal ID: Can Med Educ J
NLM Journal ID: 101560935
Journal ID: CMEJ

Canadian Medical Education Journal

EISSN: 1923-1202
Publisher: University of Saskatchewan

ARTICLE INFORMATION
==============================
Subjects: Pa - 2 – 4

12 Tips: A Guideline to Establishing a Successful Medical Education Program

Thomas Haupt Dalhousie University

Todd Dow Dalhousie University

Mike Smyth Dalhousie University

Alysha Roberts Dalhousie University

Kavita Raju Dalhousie University

Tom Toguri Dalhousie University

Anna MacLeod Dalhousie University

Electronic publication date: 2019 Apr 3

JOURNAL INFORMATION
==============================
NLM Title Abbreviation: Can Med Educ J
Journal ID: Can Med Educ J
NLM Journal ID: 101560935
Journal ID: CMEJ

Canadian Medical Education Journal

EISSN: 1923-1202
Publisher: University of Saskatchewan

ARTICLE INFORMATION
==============================
Subjects: Pa - 2 – 5

An Interactive iBook for University of Toronto Anesthesia Medical Students

Alex Sapa University of Toronto

Ahtsham Niazi University of Toronto

Clyde Matava University of Toronto

Electronic publication date: 2019 Apr 3

JOURNAL INFORMATION
==============================
NLM Title Abbreviation: Can Med Educ J
Journal ID: Can Med Educ J
NLM Journal ID: 101560935
Journal ID: CMEJ

Canadian Medical Education Journal

EISSN: 1923-1202
Publisher: University of Saskatchewan

ARTICLE INFORMATION
==============================
Subjects: Pa - 2 – 6

Development and evaluation of a new interactive genetic education module as a teaching tool in the Division of Clinical and Metabolic Genetics at the Hospital for Sick Children

Tina (Shijie) Zhou Western University

Lucie Dupuis University of Toronto

Cheryl Cytrynbaum University of Toronto

Roberto Mendoza-Londono University of Toronto

Electronic publication date: 2019 Apr 3

JOURNAL INFORMATION
==============================
NLM Title Abbreviation: Can Med Educ J
Journal ID: Can Med Educ J
NLM Journal ID: 101560935
Journal ID: CMEJ

Canadian Medical Education Journal

EISSN: 1923-1202
Publisher: University of Saskatchewan

ARTICLE INFORMATION
==============================
Subjects: Pa - 2 – 7

Preparing for Surgery Observerships during Medical School: A Cross-Sectional Study

Arielle Brickman Queen’s University

Vincent Wu University of Toronto

Boris Zevin Queen’s University

Electronic publication date: 2019 Apr 3

JOURNAL INFORMATION
==============================
NLM Title Abbreviation: Can Med Educ J
Journal ID: Can Med Educ J
NLM Journal ID: 101560935
Journal ID: CMEJ

Canadian Medical Education Journal

EISSN: 1923-1202
Publisher: University of Saskatchewan

ARTICLE INFORMATION
==============================
Subjects: Pa - 2 – 8

Exploring the impact of early pre-clerkship surgical exposure and mentorship on the perceptions of medical students towards surgical career stereotypes: A qualitative analysis

Sylvie Bowden* University of Toronto

Shawn Khan* University of Toronto

Kimia Sorouri University of Toronto

Stephanie Searle University of Toronto

Lauren Carr University of Toronto

Jory Simpson University of Toronto

Electronic publication date: 2019 Apr 3

JOURNAL INFORMATION
==============================
NLM Title Abbreviation: Can Med Educ J
Journal ID: Can Med Educ J
NLM Journal ID: 101560935
Journal ID: CMEJ

Canadian Medical Education Journal

EISSN: 1923-1202
Publisher: University of Saskatchewan

ARTICLE INFORMATION
==============================
Subjects: Pa - 3 – 1

Scoping Review on Behaviour Remediation in Post-Graduate Medical Trainees

Farina Rafiq Dalhousie University

Babar Haroon Dr. Dalhousie University

Electronic publication date: 2019 Apr 3

JOURNAL INFORMATION
==============================
NLM Title Abbreviation: Can Med Educ J
Journal ID: Can Med Educ J
NLM Journal ID: 101560935
Journal ID: CMEJ

Canadian Medical Education Journal

EISSN: 1923-1202
Publisher: University of Saskatchewan

ARTICLE INFORMATION
==============================
Subjects: Pa - 3 – 2

Challenges to the Integration of Entrustable Professional Activities into Clinical Clerkship

Zhubo Zhang Queen’s University

Avrilynn Ding Queen’s University

Andrea Winthrop Queen’s University

Eleni Katsoulas Queen’s University

Electronic publication date: 2019 Apr 3

JOURNAL INFORMATION
==============================
NLM Title Abbreviation: Can Med Educ J
Journal ID: Can Med Educ J
NLM Journal ID: 101560935
Journal ID: CMEJ

Canadian Medical Education Journal

EISSN: 1923-1202
Publisher: University of Saskatchewan

ARTICLE INFORMATION
==============================
Subjects: Pa - 3 – 3

What Does It Mean to Be Competent? An Inter-professional Definition in Anesthesia

Natalie Lidster McMaster University

Caitlin VanDeCappelle McMaster University

Saroo Sharma McMaster University

Electronic publication date: 2019 Apr 3

JOURNAL INFORMATION
==============================
NLM Title Abbreviation: Can Med Educ J
Journal ID: Can Med Educ J
NLM Journal ID: 101560935
Journal ID: CMEJ

Canadian Medical Education Journal

EISSN: 1923-1202
Publisher: University of Saskatchewan

ARTICLE INFORMATION
==============================
Subjects: Pa - 3 – 4

The use of simulation in emergency medicine UGME clerkship education: A QI Initiative

Samuel Wilson University of Ottawa

Dalia Karol University of Ottawa

Christopher G. Elliott University of Ottawa

Kuan-chin Jean Chen University of Ottawa

Electronic publication date: 2019 Apr 3

JOURNAL INFORMATION
==============================
NLM Title Abbreviation: Can Med Educ J
Journal ID: Can Med Educ J
NLM Journal ID: 101560935
Journal ID: CMEJ

Canadian Medical Education Journal

EISSN: 1923-1202
Publisher: University of Saskatchewan

ARTICLE INFORMATION
==============================
Subjects: Pa - 3 – 5

Developing a simulation curriculum to teach medical students to perform an ultrasound-guided needle insertion

Sachin Pasricha Queen’s University

Zsuzsanna Keri Queen’s University

Matthew Holden Queen’s University

Tamas Ungi Queen’s University

Gabor Fichtinger Queen’s University

Electronic publication date: 2019 Apr 3

JOURNAL INFORMATION
==============================
NLM Title Abbreviation: Can Med Educ J
Journal ID: Can Med Educ J
NLM Journal ID: 101560935
Journal ID: CMEJ

Canadian Medical Education Journal

EISSN: 1923-1202
Publisher: University of Saskatchewan

ARTICLE INFORMATION
==============================
Subjects: Pa - 3 – 6

Standardized patients no more: using non-standardized patient stations in a clerkship objective structured clinical examination

Vijay Sandhu University of Toronto

Raed Hawa University of Toronto

Carla Garcia University of Toronto

Andrea Waddell University of Toronto

Electronic publication date: 2019 Apr 3

JOURNAL INFORMATION
==============================
NLM Title Abbreviation: Can Med Educ J
Journal ID: Can Med Educ J
NLM Journal ID: 101560935
Journal ID: CMEJ

Canadian Medical Education Journal

EISSN: 1923-1202
Publisher: University of Saskatchewan

ARTICLE INFORMATION
==============================
Subjects: Pa - 3 – 7

Development of an Exercise During Pregnancy Online Learning Module for Family Medicine Residents

Sophie Glanz University of Toronto

Rachel McDonald University of Toronto

Lisa Kachaniwsky University of Toronto

Sabrina Kolker University of Toronto

Pearl Yang University of Toronto

Karen Fleming University of Toronto

Electronic publication date: 2019 Apr 3

JOURNAL INFORMATION
==============================
NLM Title Abbreviation: Can Med Educ J
Journal ID: Can Med Educ J
NLM Journal ID: 101560935
Journal ID: CMEJ

Canadian Medical Education Journal

EISSN: 1923-1202
Publisher: University of Saskatchewan

ARTICLE INFORMATION
==============================
Subjects: Pa 3–8

How a medical students committee drastically changed the way to prepare for OSCE through peer education methods

Vincent Ménard-Cholette Université Laval

Élizabeth Richard Université Laval

Julie Thériault Université Laval

Emily Wang Université Laval

Electronic publication date: 2019 Apr 3

JOURNAL INFORMATION
==============================
NLM Title Abbreviation: Can Med Educ J
Journal ID: Can Med Educ J
NLM Journal ID: 101560935
Journal ID: CMEJ

Canadian Medical Education Journal

EISSN: 1923-1202
Publisher: University of Saskatchewan

ARTICLE INFORMATION
==============================
Subjects: Pa 3–9

Using Self-Reported Measures of Confidence and Anxiety to Determine the Efficacy of the Surgical Exploration And Discovery (SEAD) Program in Reducing Anxiety and Increasing Confidence in Performing Procedural Skills

Frank Battaglia University of Ottawa

Emilie Langlois University of Ottawa

Marisa Market University of Ottawa

John Shin University of Ottawa

Christine Seabrook University of Ottawa

Tim Brandys University of Ottawa

Electronic publication date: 2019 Apr 3

JOURNAL INFORMATION
==============================
NLM Title Abbreviation: Can Med Educ J
Journal ID: Can Med Educ J
NLM Journal ID: 101560935
Journal ID: CMEJ

Canadian Medical Education Journal

EISSN: 1923-1202
Publisher: University of Saskatchewan

ARTICLE INFORMATION
==============================
Subjects: Pa - 4 – 1

Quick Refresher Sessions (QRS): improving chest compression training for medical students

Alexander Cormier Queen’s University

Erin Brennan Queen’s University

Electronic publication date: 2019 Apr 3

JOURNAL INFORMATION
==============================
NLM Title Abbreviation: Can Med Educ J
Journal ID: Can Med Educ J
NLM Journal ID: 101560935
Journal ID: CMEJ

Canadian Medical Education Journal

EISSN: 1923-1202
Publisher: University of Saskatchewan

ARTICLE INFORMATION
==============================
Subjects: Pa - 4 – 2

Traditional versus 360-Degree Video- Modelling for teaching Central Venous Catheter placement using task trainers

Evan Mah University of Saskatchewan

Megan Deck University of Saskatchewan

Julie Yu University of Saskatchewan

Kish Lyster University of Saskatchewan

Joann Kawchuk University of Saskatchewan

Brian Brownbridge University of Saskatchewan

Alison Turnquist University of Saskatchewan

Luke Terrett University of Saskatchewan

Brent Thoma University of Saskatchewan

Electronic publication date: 2019 Apr 3

JOURNAL INFORMATION
==============================
NLM Title Abbreviation: Can Med Educ J
Journal ID: Can Med Educ J
NLM Journal ID: 101560935
Journal ID: CMEJ

Canadian Medical Education Journal

EISSN: 1923-1202
Publisher: University of Saskatchewan

ARTICLE INFORMATION
==============================
Subjects: Pa - 4 – 4

A Pre-Clerkship Procedural Curriculum designed for the future of Canadian Medical Education: A Pilot and Feasibility Study

Frank Battaglia University of Ottawa

Maria Merlano University of Ottawa

Céline Sayed University of Ottawa

Meghan McConnell University of Ottawa

Christopher Ramnanan University of Ottawa

Nikhil Rastogi University of Ottawa

Electronic publication date: 2019 Apr 3

JOURNAL INFORMATION
==============================
NLM Title Abbreviation: Can Med Educ J
Journal ID: Can Med Educ J
NLM Journal ID: 101560935
Journal ID: CMEJ

Canadian Medical Education Journal

EISSN: 1923-1202
Publisher: University of Saskatchewan

ARTICLE INFORMATION
==============================
Subjects: Pa - 4 – 5

Program Assessment: Taking stock of the current state of Canadian Undergraduate Medical Education in Procedural Skills Curricula

Frank Battaglia University of Ottawa

Meghan McConnell University of Ottawa

Christopher Ramnanan University of Ottawa

Nikhil Rastogi University of Ottawa

Maria Merlano University of Ottawa

Celine Sayed University of Ottawa

Electronic publication date: 2019 Apr 3

JOURNAL INFORMATION
==============================
NLM Title Abbreviation: Can Med Educ J
Journal ID: Can Med Educ J
NLM Journal ID: 101560935
Journal ID: CMEJ

Canadian Medical Education Journal

EISSN: 1923-1202
Publisher: University of Saskatchewan

ARTICLE INFORMATION
==============================
Subjects: Pa - 4 – 6

"Top points for bedside manner": Evaluating Inpatient Volunteer Satisfaction with Pre-Clerkship Clinical History and Physical Examination Training

Michael Elfassy University of Toronto

Laura Duncan University of Toronto

Alison Green University of Toronto

Katina Tzanetos University of Toronto

Joyce Nyhof-Young University of Toronto

Electronic publication date: 2019 Apr 3

JOURNAL INFORMATION
==============================
NLM Title Abbreviation: Can Med Educ J
Journal ID: Can Med Educ J
NLM Journal ID: 101560935
Journal ID: CMEJ

Canadian Medical Education Journal

EISSN: 1923-1202
Publisher: University of Saskatchewan

ARTICLE INFORMATION
==============================
Subjects: Pa - 4 – 7

Developing Skills for Developmental Disabilities in Preclinical Medical Students: Identifying How Adjuvant Clinical Electives can Impact Student Confidence

Alexis Fong-Leboeuf University of Alberta

Irina Simin University of Alberta

Andy Le University of Alberta

Debra Andrews University of Alberta

Electronic publication date: 2019 Apr 3

JOURNAL INFORMATION
==============================
NLM Title Abbreviation: Can Med Educ J
Journal ID: Can Med Educ J
NLM Journal ID: 101560935
Journal ID: CMEJ

Canadian Medical Education Journal

EISSN: 1923-1202
Publisher: University of Saskatchewan

ARTICLE INFORMATION
==============================
Subjects: Pb - 1 – 1

Enquête de terrain sur la conformité des activités de DPC aux normes d'accréditation : un outil d'amélioration continue

Cynthia Henriksen Université de Montréal

Vincent Jobin Université de Montréal

Louise Fugère Université de Montréal

Electronic publication date: 2019 Apr 3

JOURNAL INFORMATION
==============================
NLM Title Abbreviation: Can Med Educ J
Journal ID: Can Med Educ J
NLM Journal ID: 101560935
Journal ID: CMEJ

Canadian Medical Education Journal

EISSN: 1923-1202
Publisher: University of Saskatchewan

ARTICLE INFORMATION
==============================
Subjects: Pb - 1 – 2

MD Program Teaching Awards of Excellence (MPTA): Celebrating pedagogical excellence in undergraduate medicine

Flora Jung University of Toronto

Mike Wiley University of Toronto

Frazer Howard University of Toronto

Nicholas Sequeira University of Toronto

Joyce Nyhof-Young The College of Family Physicians of Canada

Electronic publication date: 2019 Apr 3

JOURNAL INFORMATION
==============================
NLM Title Abbreviation: Can Med Educ J
Journal ID: Can Med Educ J
NLM Journal ID: 101560935
Journal ID: CMEJ

Canadian Medical Education Journal

EISSN: 1923-1202
Publisher: University of Saskatchewan

ARTICLE INFORMATION
==============================
Subjects: Pb - 1 – 3

Participant outcomes of student-run interprofessional health promotion services to vulnerable populations in Sudbury, Ontario

Sarah Mavin Northern Ontario School of Medicine

Gayle Adams-Carpino Northern Ontario School of Medicine

April Kindrat Northern Ontario School of Medicine

Electronic publication date: 2019 Apr 3

JOURNAL INFORMATION
==============================
NLM Title Abbreviation: Can Med Educ J
Journal ID: Can Med Educ J
NLM Journal ID: 101560935
Journal ID: CMEJ

Canadian Medical Education Journal

EISSN: 1923-1202
Publisher: University of Saskatchewan

ARTICLE INFORMATION
==============================
Subjects: Pb - 1 – 4

An Unexplored Landscape - Intra- Professional Configurations and Patient Care Networks

Katherina Baranova Western University

Jacqueline Torti Western University

Mark Goldszmidt Western University

Electronic publication date: 2019 Apr 3

JOURNAL INFORMATION
==============================
NLM Title Abbreviation: Can Med Educ J
Journal ID: Can Med Educ J
NLM Journal ID: 101560935
Journal ID: CMEJ

Canadian Medical Education Journal

EISSN: 1923-1202
Publisher: University of Saskatchewan

ARTICLE INFORMATION
==============================
Subjects: Pb - 1 – 5

A Health Professional (HP) Student-Led Approach to Building Interprofessional Collaborative Practice (IPCP) Skills

Maichael Thejoe University of British Columbia

Carrie Krekoski University of British Columbia

Christie Newton University of British Columbia

Electronic publication date: 2019 Apr 3

JOURNAL INFORMATION
==============================
NLM Title Abbreviation: Can Med Educ J
Journal ID: Can Med Educ J
NLM Journal ID: 101560935
Journal ID: CMEJ

Canadian Medical Education Journal

EISSN: 1923-1202
Publisher: University of Saskatchewan

ARTICLE INFORMATION
==============================
Subjects: Pb - 1 – 6

Lost in translation: How informative are residents' responses to care team queries through a mobile-based text messaging system?

Nicholas Sequeira University of Toronto

Tom MacMillan University of Toronto

Robert Wu University of Toronto

Rodrigo Cavalcanti University of Toronto

Electronic publication date: 2019 Apr 3

JOURNAL INFORMATION
==============================
NLM Title Abbreviation: Can Med Educ J
Journal ID: Can Med Educ J
NLM Journal ID: 101560935
Journal ID: CMEJ

Canadian Medical Education Journal

EISSN: 1923-1202
Publisher: University of Saskatchewan

ARTICLE INFORMATION
==============================
Subjects: Pb - 1 – 7

CHIUS: Examining the Impact of Student- Run Clinics on Interprofessional Healthcare Students

Mona Maleki University of British Columbia

Kelly Huang University of British Columbia

Heather McEwen University of British Columbia

Glenn Regehr University of British Columbia

Electronic publication date: 2019 Apr 3

JOURNAL INFORMATION
==============================
NLM Title Abbreviation: Can Med Educ J
Journal ID: Can Med Educ J
NLM Journal ID: 101560935
Journal ID: CMEJ

Canadian Medical Education Journal

EISSN: 1923-1202
Publisher: University of Saskatchewan

ARTICLE INFORMATION
==============================
Subjects: Pb - 2 – 1

Implementing exercise as medicine into undergraduate medical curricula

Irina Simin University of Alberta

Dhiren Naidu University of Alberta

Tracey Hillier University of Alberta

Electronic publication date: 2019 Apr 3

JOURNAL INFORMATION
==============================
NLM Title Abbreviation: Can Med Educ J
Journal ID: Can Med Educ J
NLM Journal ID: 101560935
Journal ID: CMEJ

Canadian Medical Education Journal

EISSN: 1923-1202
Publisher: University of Saskatchewan

ARTICLE INFORMATION
==============================
Subjects: Pb - 2 – 2

Health Policy and Healthcare System Sciences: An Essential Component of Medical Education

Tul-Zahra Rida University of Ottawa

Laura Muldoon University of Ottawa

Marie-Hélène Chomienne* University of Ottawa

Karine Toupin-April* University of Ottawa

Electronic publication date: 2019 Apr 3

JOURNAL INFORMATION
==============================
NLM Title Abbreviation: Can Med Educ J
Journal ID: Can Med Educ J
NLM Journal ID: 101560935
Journal ID: CMEJ

Canadian Medical Education Journal

EISSN: 1923-1202
Publisher: University of Saskatchewan

ARTICLE INFORMATION
==============================
Subjects: Pb - 2 – 3

Designing a Medical Education Program to teach Pediatric Palliative Care to Health Care Professionals

Chloe Thabet University of Ottawa

Megan Doherty Dr. University of Ottawa

Electronic publication date: 2019 Apr 3

JOURNAL INFORMATION
==============================
NLM Title Abbreviation: Can Med Educ J
Journal ID: Can Med Educ J
NLM Journal ID: 101560935
Journal ID: CMEJ

Canadian Medical Education Journal

EISSN: 1923-1202
Publisher: University of Saskatchewan

ARTICLE INFORMATION
==============================
Subjects: Pb - 2 – 4

Student experiences with on-line modular patient safety and quality improvement curriculum

Tatiana Fras University of Saskatchewan

Meredith McKague Dr. University of Saskatchewan

Regina Taylor-Gjevre Dr. University of Saskatchewan

Michael Prystajecky Dr University of Saskatchewan

Krista Trinder University of Saskatchewan

Electronic publication date: 2019 Apr 3

JOURNAL INFORMATION
==============================
NLM Title Abbreviation: Can Med Educ J
Journal ID: Can Med Educ J
NLM Journal ID: 101560935
Journal ID: CMEJ

Canadian Medical Education Journal

EISSN: 1923-1202
Publisher: University of Saskatchewan

ARTICLE INFORMATION
==============================
Subjects: Pb - 2 – 5

Piloting Photovoice in a Student-Run Clinic: Implementing a novel reflection method with interdisciplinary healthcare trainees during service-based learning placements

Fangyi Liu University of Toronto

Yang Qin University of Toronto

Imaan Javeed University of Toronto

Joyce Nyhof-Young University of Toronto

Electronic publication date: 2019 Apr 3

JOURNAL INFORMATION
==============================
NLM Title Abbreviation: Can Med Educ J
Journal ID: Can Med Educ J
NLM Journal ID: 101560935
Journal ID: CMEJ

Canadian Medical Education Journal

EISSN: 1923-1202
Publisher: University of Saskatchewan

ARTICLE INFORMATION
==============================
Subjects: Pb - 2 – 6

Discovery Healthcare: Promoting Interest in Healthcare Careers among High School Students from South-Western Ontario

Vivian Tia Western University

Richard Yu Western University

John Cameron Western University

Dylan Russelo Western University

Danny Kim Western University

Electronic publication date: 2019 Apr 3

JOURNAL INFORMATION
==============================
NLM Title Abbreviation: Can Med Educ J
Journal ID: Can Med Educ J
NLM Journal ID: 101560935
Journal ID: CMEJ

Canadian Medical Education Journal

EISSN: 1923-1202
Publisher: University of Saskatchewan

ARTICLE INFORMATION
==============================
Subjects: Pb - 2 – 7

Prescribing Competency in Undergraduate Medical Education

Alison Bell Queen’s University

Michelle Gibson Queen’s University

Electronic publication date: 2019 Apr 3

JOURNAL INFORMATION
==============================
NLM Title Abbreviation: Can Med Educ J
Journal ID: Can Med Educ J
NLM Journal ID: 101560935
Journal ID: CMEJ

Canadian Medical Education Journal

EISSN: 1923-1202
Publisher: University of Saskatchewan

ARTICLE INFORMATION
==============================
Subjects: Pb - 2 – 8

"Are You Post-Call?": Promoting safe transport after call shifts

Yasamin Mahjoub University of Alberta

Miranda Wan University of Alberta

Marie DeCock University of Alberta

Melanie Lewis University of Alberta

Electronic publication date: 2019 Apr 3

JOURNAL INFORMATION
==============================
NLM Title Abbreviation: Can Med Educ J
Journal ID: Can Med Educ J
NLM Journal ID: 101560935
Journal ID: CMEJ

Canadian Medical Education Journal

EISSN: 1923-1202
Publisher: University of Saskatchewan

ARTICLE INFORMATION
==============================
Subjects: Pb - 2 – 9

What causes pediatric prescribing errors? Studying practice to guide education

Richard Conn Queen's University Belfast

Orla Kearney Queen's University Belfast

Michael Shields Queen's University Belfast

Mary Tully University of Manchester

Tim Dornan Queen's University Belfast

Electronic publication date: 2019 Apr 3

JOURNAL INFORMATION
==============================
NLM Title Abbreviation: Can Med Educ J
Journal ID: Can Med Educ J
NLM Journal ID: 101560935
Journal ID: CMEJ

Canadian Medical Education Journal

EISSN: 1923-1202
Publisher: University of Saskatchewan

ARTICLE INFORMATION
==============================
Subjects: Pb - 3 – 1

Gender disparities in academic reference letters: A systematic review

Shawn Khan University of Toronto

Abirami Kirubarajan* University of Toronto

Tahmina Shamsheri* McMaster University

Sangeeta Mehta University of Toronto

Electronic publication date: 2019 Apr 3

JOURNAL INFORMATION
==============================
NLM Title Abbreviation: Can Med Educ J
Journal ID: Can Med Educ J
NLM Journal ID: 101560935
Journal ID: CMEJ

Canadian Medical Education Journal

EISSN: 1923-1202
Publisher: University of Saskatchewan

ARTICLE INFORMATION
==============================
Subjects: Pb - 3 – 2

The Indigenous Program of the University of Sherbrooke Faculty of Medicine and Health Sciences

Mélissa Nepton-Riverin Université de Sherbrooke

Genevieve Lavoie Université de Sherbrooke

Émilie Courtois Université de Sherbrooke

Pascale Ouellet- Dufour Université de Sherbrooke

Electronic publication date: 2019 Apr 3

JOURNAL INFORMATION
==============================
NLM Title Abbreviation: Can Med Educ J
Journal ID: Can Med Educ J
NLM Journal ID: 101560935
Journal ID: CMEJ

Canadian Medical Education Journal

EISSN: 1923-1202
Publisher: University of Saskatchewan

ARTICLE INFORMATION
==============================
Subjects: Pb - 3 – 3

Evaluating a Novel Integrated Clinical Skills Program for Undergraduate Medical Students

Kaitlyn Lam University of Toronto

Ben Shachar University of Toronto

Fok-Han Leung University of Toronto

David Rojas Gualdron University of Toronto

Joyce Nyhof-Young University of Toronto

Electronic publication date: 2019 Apr 3

JOURNAL INFORMATION
==============================
NLM Title Abbreviation: Can Med Educ J
Journal ID: Can Med Educ J
NLM Journal ID: 101560935
Journal ID: CMEJ

Canadian Medical Education Journal

EISSN: 1923-1202
Publisher: University of Saskatchewan

ARTICLE INFORMATION
==============================
Subjects: Pb - 3 – 4

Evaluation of the effectiveness of the Global Medical Student Partnership (GMSP) initiative in medical education

Hannah Samuels University of Toronto

Vanessa Rojas Luengas University of Toronto

Arman Zereshkian University of Toronto

Paula Veinot University of Toronto

Ashna Bowry University of Toronto

Marcus Law University of Toronto

Electronic publication date: 2019 Apr 3

JOURNAL INFORMATION
==============================
NLM Title Abbreviation: Can Med Educ J
Journal ID: Can Med Educ J
NLM Journal ID: 101560935
Journal ID: CMEJ

Canadian Medical Education Journal

EISSN: 1923-1202
Publisher: University of Saskatchewan

ARTICLE INFORMATION
==============================
Subjects: Pb - 3 – 5

Using Think-Aloud Method to Evaluate & Re-Design the University of Alberta MD Program Website

Aashna Duggal University of Alberta

Thomas Jeffery University of Alberta

Dr. Joanne Rodger University of Alberta

Patrick Von Hauff University of Alberta

Dr. Tracey Hillier University of Alberta

Electronic publication date: 2019 Apr 3

JOURNAL INFORMATION
==============================
NLM Title Abbreviation: Can Med Educ J
Journal ID: Can Med Educ J
NLM Journal ID: 101560935
Journal ID: CMEJ

Canadian Medical Education Journal

EISSN: 1923-1202
Publisher: University of Saskatchewan

ARTICLE INFORMATION
==============================
Subjects: Pb - 3 – 6

Perceptions around duty hour restrictions in residency: A qualitative study of online Canadian medical student discussion groups

Anahita Dehmoobad Sharifabadi University of Ottawa

Asif Doja University of Ottawa

Chantalle Clarkin Bruyère Research Institute, 43 Bruyère St. Ottawa, Ontario, K1N 5C8

Electronic publication date: 2019 Apr 3

JOURNAL INFORMATION
==============================
NLM Title Abbreviation: Can Med Educ J
Journal ID: Can Med Educ J
NLM Journal ID: 101560935
Journal ID: CMEJ

Canadian Medical Education Journal

EISSN: 1923-1202
Publisher: University of Saskatchewan

ARTICLE INFORMATION
==============================
Subjects: Pb - 3 – 7

Training Medical Students and Residents in the Use of Electronic Health Records: A Systematic Review of the Literature

Akshay Rajaram Queen’s University

Nimesh Patel Queen’s University

Zachary Hickey Queen’s University

Joseph Newbigging Queen’s University

Brent Wolfrom Queen’s University

Electronic publication date: 2019 Apr 3

JOURNAL INFORMATION
==============================
NLM Title Abbreviation: Can Med Educ J
Journal ID: Can Med Educ J
NLM Journal ID: 101560935
Journal ID: CMEJ

Canadian Medical Education Journal

EISSN: 1923-1202
Publisher: University of Saskatchewan

ARTICLE INFORMATION
==============================
Subjects: Pb - 4 – 1

Project ECHO: Filling the Gap in Pediatric Palliative Care Training in India

Emily Evans University of Ottawa

Megan Doherty Children's Hospital of Eastern Ontario

Electronic publication date: 2019 Apr 3

JOURNAL INFORMATION
==============================
NLM Title Abbreviation: Can Med Educ J
Journal ID: Can Med Educ J
NLM Journal ID: 101560935
Journal ID: CMEJ

Canadian Medical Education Journal

EISSN: 1923-1202
Publisher: University of Saskatchewan

ARTICLE INFORMATION
==============================
Subjects: Pb - 4 – 2

Pilot Project: Implementation of Deaf Cultural Competency Training in the McGill Faculty of Medicine's Undergraduate Medical Curriculum

Aselin Weng McGill

Brian Tran McGill

Electronic publication date: 2019 Apr 3

JOURNAL INFORMATION
==============================
NLM Title Abbreviation: Can Med Educ J
Journal ID: Can Med Educ J
NLM Journal ID: 101560935
Journal ID: CMEJ

Canadian Medical Education Journal

EISSN: 1923-1202
Publisher: University of Saskatchewan

ARTICLE INFORMATION
==============================
Subjects: Pb - 4 – 3

Does personality matter? Perceptions of introverts in general surgery

Victoria Luong Acadia University

Chris Shields Acadia University

Allison Petrie Acadia University

Katerina Neumann Dalhousie University

Electronic publication date: 2019 Apr 3

JOURNAL INFORMATION
==============================
NLM Title Abbreviation: Can Med Educ J
Journal ID: Can Med Educ J
NLM Journal ID: 101560935
Journal ID: CMEJ

Canadian Medical Education Journal

EISSN: 1923-1202
Publisher: University of Saskatchewan

ARTICLE INFORMATION
==============================
Subjects: Pb - 4 – 4

A Systematic Review of Online Academic Resources: Renal and Genitourinary

Andy Grock

Anuja Bhalerao University of Toronto

Teresa Chan McMaster University

Brent Thoma University of Saskatchewan

Seth Trueger

Electronic publication date: 2019 Apr 3

JOURNAL INFORMATION
==============================
NLM Title Abbreviation: Can Med Educ J
Journal ID: Can Med Educ J
NLM Journal ID: 101560935
Journal ID: CMEJ

Canadian Medical Education Journal

EISSN: 1923-1202
Publisher: University of Saskatchewan

ARTICLE INFORMATION
==============================
Subjects: Pb - 4 – 5

Fostering adaptive expertise: exploring guideline use in mental health care

Natasha Tang Queen’s University

Maria Mylopoulus University of Toronto

Sanjeev Sockalingam University of Toronto

Electronic publication date: 2019 Apr 3

JOURNAL INFORMATION
==============================
NLM Title Abbreviation: Can Med Educ J
Journal ID: Can Med Educ J
NLM Journal ID: 101560935
Journal ID: CMEJ

Canadian Medical Education Journal

EISSN: 1923-1202
Publisher: University of Saskatchewan

ARTICLE INFORMATION
==============================
Subjects: Pb - 4 – 7

Virtual Doc: A virtual reality simulation teaching paediatric cardiopulmonary resuscitation

Janaya E. Perron University of New South Wales

Michael J. Coffey University of New South Wales

Andrew Lovell-Simons University of New South Wales

Zheyu Li University of New South Wales

Seiya Takeda University of New South Wales

Luis Dominguez University of New South Wales

Chee Y. Ooi University of New South Wales

Electronic publication date: 2019 Apr 3

JOURNAL INFORMATION
==============================
NLM Title Abbreviation: Can Med Educ J
Journal ID: Can Med Educ J
NLM Journal ID: 101560935
Journal ID: CMEJ

Canadian Medical Education Journal

EISSN: 1923-1202
Publisher: University of Saskatchewan

ARTICLE INFORMATION
==============================
Subjects: Pb - 4 – 8

VR Plexus: Can virtual reality on a smartphone improve neurology learning amongst medical students?

Maxwell Ng McMaster University

Rohit Malyala McMaster University

He Tian Chen McMaster University

Katherine Kuo McMaster University

Alexander K. Ball McMaster University

Michel P. Rathbone McMaster University

Bruce Wainman McMaster University

Electronic publication date: 2019 Apr 3

